# Challenges in addiction-affected families: a systematic review of qualitative studies

**DOI:** 10.1186/s12888-023-04927-1

**Published:** 2023-06-16

**Authors:** Mostafa Mardani, Fardin Alipour, Hassan Rafiey, Masoud Fallahi-Khoshknab, Maliheh Arshi

**Affiliations:** 1 Department of Social Work, University of Social Welfare and Rehabilitation Sciences, Tehran, Iran; 2 Department of Social Work, Social Welfare Management Research Center, Social Health Institute, University of Social Welfare and Rehabilitation Sciences, Tehran, Iran; 3 Social Welfare Management Research Center, University of Social Welfare and Rehabilitation Sciences, Tehran, Iran; 4 Department of Nursing, University of Social Welfare and Rehabilitation Sciences, Tehran, Iran

**Keywords:** Addiction, Addiction-affected family, Drug user, Systematic review, Qualitative research, Thematic analysis

## Abstract

**Background:**

The relative paucity of research on Addiction-Affected Families’ (AAF) issues and the lack of attention given to their difficulties and treatment in interventions and clinical practices indicate that the primary focus consistently revolves around individuals with addictive disorders, even when the treatment process involves their families. However, it is believed that family members endure significant pressures that result in extensive negative consequences on the personal, familial, and social aspects of their lives. Aiming for a better understanding of the challenges and issues that AAF’s experience, this systematic review explored qualitative studies with a focus on the impact of addiction on different aspects of families.

**Methods:**

We searched Research Gate, Scopus, Web of Science, ProQuest, Elsevier, and Google Scholar Databases. We included studies of qualitative design which have investigated the effects of addiction on families. Non-English language studies, medical views, and quantitative approaches were excluded. Participants in the selected studies included parents, children, couples, sisters/brothers, relatives, drug users and specialists. The data from the selected studies were extracted using a standard format for the systematic review of qualitative research (the National Institute of Health and Care Excellence [NICE] 2102a).

**Results:**

A thematic analysis of the findings of the studies identified 5 main themes: 1) initial shock (family encounter, searching for why), 2) family in the fog (social isolation, stigma and label), 3) sequence of disorders (emotional decline, negative behavioral experiences, mental disturbance, physical degeneration, family burden), 4) internal family chaos (instability of relationships, shadow people, erosive confrontation with the drug-using member, a newly emerging member, collapsing system, financial collapse), and 5) self-protection (attracting information, support, and protective sources, coping and adjusting the effects, the emergence of spirituality).

**Conclusion:**

This systematic review of qualitative research highlights the various and complex issues which addiction-affected families go through in terms of financial, social, cultural, mental and physical health problems, as a result of which experts of the field are needed to investigate and take measures. The findings can inform policy and practice and the development of interventions aimed to lighten the burdens which addiction-affected families carry.

## Background

Today, due to the expansion of societies, the emergence of various social groups, and the diversity of needs, countries and the general public have faced a new world of needs, interests, progress, and even various problems. These issues and problems have penetrated the spirit of societies and have influenced individuals’ social world. In addition to the effects on the individuals, such problems have also had widespread effects on larger levels, such as the family and society levels.

When a family member enters the cycle of problems, the family, as the first group and institution, starts its support services for the individual, and since, in most cases, the family and its members do not have sufficient and specialized knowledge about the individual’s problem, they are under too much pressure. Maintenance, care, and support of a member with an injury and problem put a heavy burden on the shoulders of families, and since some families are not able to adapt to the problem and react appropriately to it, the family’s normal system and functioning are disturbed, and the family deviates from its normal path due to the severity of the problem [1, 2]. Addiction is one of the biggest social problems that the current world is facing. It is a problem that does not only involve the drug user but also affects several individuals and social environments around the person [3, 4]. Studies have shown that the impacts of drug and alcohol use on families are undeniable [5–7] and exposes family members to a wide range of issues such as: socio-economic and mental health problems, abuse, conflicts, dysfunctional family structure, various issues in community life, and several problems related to married life [8]. It is noteworthy that in the absence of sufficient social and governmental support, the burden of addiction or any other problem will be quite heavy and stressful for families, which, in addition to affecting the structure as well as the function and role of the family, makes family members face various and severe pressures and stresses [9–11]. Of course, it is essential to mention that the various effects of addiction in different cultures and also the drug user’s role in the family (wife, parent, or child) are not to be ignored [12]. Although such families suffer from the same pain, the severity of the challenges they face vary in different cultures [13].

Despite the abundance of research on addiction and its implications for individuals, there is a dearth of comprehensive understanding regarding the distinctive challenges faced by families affected by addiction. Although studies have provided valuable insights into the personal experiences of individuals grappling with addiction, a substantial gap still exists in the current literature when it comes to exploring the specific challenges, dynamics, and coping mechanisms within families affected by addiction [14, 15]. In other words, such studies fail to notice the other side of addiction, which is addiction-affected families [16].

In addition, those other studies which do in fact investigate the challenges faced by addiction-affected families, have not comprehensively examined them and have targeted only limited parts of AAF’s experiences. For instance, in one study, only certain single challenges in isolation was considered [17], and in another paper, the effect of relationship, social and cultural factors on the AAF’s experiences were investigated [13]. Furthermore, the only existing qualitative review in this field reviews the qualitative studies on addiction-affected families until the year 2010 [18]. This is while the challenges faced by such families in recent years are beyond the ones investigated in these studies, and a comprehensive view on the problems faced by this group of people in all dimensions is still missing.

For these reasons, further research is needed in the field of AAF, so that they can ultimately lead to a change in the perspective of therapeutic measures and theoretical models in this field. By conducting comprehensive and detailed investigations a deeper understanding of AAF can be attained, which in turn has the capacity to reshape current perspectives and contribute to the advancement of this field [19]. The purpose of the present systematic review is to identify and gain a comprehensive view of qualitative studies and gain insights into the similarities and differences existing in the shared human experience of the same phenomenon through evaluation, critical analysis, and synthesis of qualitative results based on observations and main concepts in order to use the obtained data to make it possible to provide services and interventions in the area of ‘rehabilitation of addiction-affected families’. Since families play a significant role in the treatment process and a comprehensive study on the challenges that families face and the interventions and treatment processes which are most effective for them has not yet been carried out, the present study can pave the way to respond to the above-mentioned conditions.

### The present study

What distinguishes qualitative research from quantitative research is their ways of looking at various phenomena and searching methods. In other words, qualitative research seeks to investigate the experiences of addiction-affected families regarding the addiction of one of their family members and subsequently gain deep insight into the phenomenon in question. In this way, qualitative research provides understanding and insight regarding the effects of addiction on families by examining the thoughts and feelings of the participants and analyzing the extracted themes [20]. The main goal is to provide a comprehensive understanding of the differences in human experiences regarding a phenomenon by analyzing and reviewing texts, images, and interviews. Qualitative research provides valuable insight into phenomena, policies, and practices, although such research traditionally had no place in systematic reviews [21]. The importance of developing a client-based policy was internationally accepted and recognized by clients themselves. Paying attention to the voice of clients means giving them enough power to express their issues and problems (having a voice) [22]. Addiction-affected families are often isolated and are somehow service receivers due to the many challenges and problems they face, and this makes them choose different ways to reach a solution, and on this path, they come across multiple issues and difficulties; as a result, if we want to achieve a correct and integrated understanding of the problems of addiction-affected families, it is necessary to conduct qualitative research. The main focus of this systematic review is to provide a general and comprehensive view of addiction-affected families and the issues and problems this group have when confronting the drug-using member. In particular, the present study aims to identify the gaps and analyze the issues and themes from different types of qualitative research to be aware of the services and actions needed for addiction-affected families in different dimensions.

## Methods

### Search strategy and criteria

The search strategy was restricted to studies published in English regarding the effects of addiction on families published between 1990 and 2022. In the search strategy, Research Gate, Scopus, Web of Science, ProQuest, Elsevier, and Google Scholar databases were used and analyzed from 1990 to January 2022. The keywords selected for international databases included: Addiction-affected family, the impact of addiction, addiction and drug abusers, Impact of addiction on families, negative consequence of addiction on families, addiction and family. The papers were first reviewed based on the titles and abstracts. In order to identify relevant studies for the present systematic review, inclusion and exclusion criteria were considered. For this review, a three-stage selection process was used to apply the inclusion and exclusion criteria [23]: 1- Looking at the title, 2- examining the abstract to identify its association with the research question and method, and 3- reviewing the entire paper.

Based on titles and abstracts, papers were excluded if they did not explore the experiences of addiction affected families or the effects of addiction on family members; exploration of AAF’s experiences and the effects of addiction on family members was required to be either an aim of the study or a substantial finding in the results. To ensure an in-depth understanding and a rich description of experiences, only studies presenting primary data using qualitative methods were included. Mixed-method studies were included if qualitative findings were presented separately. Searches were limited to publications in the English language. Studies were excluded, if papers were restricted to individuals with/suffering from addictive disorders, and if papers were related to family factors of addiction. These exclusion criteria were introduced in order to ensure that experiences and views were current and related to the target group’s (AAF) experience. Any book chapters, Interventions, commentaries, letters, reviews, first-person accounts, and abstracts were excluded. In addition to the mentioned cases, the reference lists of the obtained studies were also examined to identify the studies that were not obtained using the above methods.

**Fig.1:**
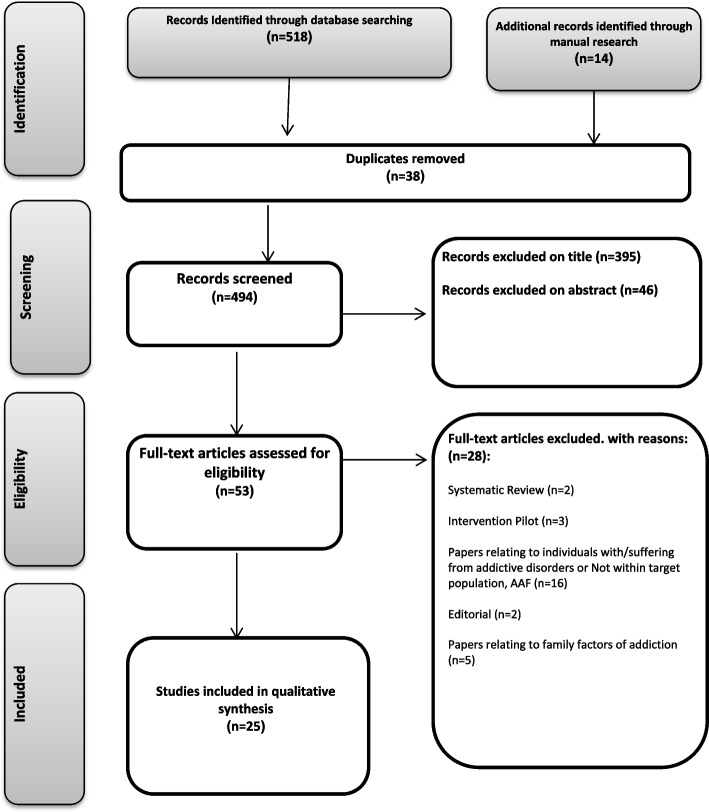
PRISMA flow diagram

In cases where the researcher was not certain about the inclusion criteria of an article, the intended article was kept for the next screening stage. Based on the search strategy of the texts, 518 studies were initially identified. An additional manual Google search was performed at this stage, and 14 more studies were identified. After removing duplicate cases and reviewing the titles and abstracts of these studies, 479 cases were removed and 53 cases were assigned to determine the inclusion criteria. After a removal process, a total of 25 studies were selected as eligible studies (Table 1). An overview of the search steps can be observed in the PRISMA flowchart (Fig. 1). The 25 studies include two studies with a combined method, and one International report of a research project whose results of the qualitative thematic analysis contain valuable data in the research process.

**Table 1:** Summary of included studies

**Studies**	**Location**	**Method**	**Data/Participants**	**Focus of Study**	**Quality**	**Limitations**
Wiarsih et al. [24]	Indonesia	Phenomenological analysis of qualitative interviews.	7 In-depth interviews with Addiction Affected Family members using the snowball sampling method	To explore experiences of families with drug-using children	+ +	The interviews were only with parents. The periods that the children used drugs and the health services accessed by families should have been identified.
McCann et al. [25]	Australia	Interpretive Phenomenological Analysis of a qualitative interviews	31 Semi-structured interviews with Addiction Affected Family members using the Purposive sampling method (14 parents, 13 couples, 4 siblings).	To understand affected family members- AFMs’ experience of aggression and violence while supporting a member with PSU, and to explicate the strategies they used to prevent and cope with this behavior.	+ +	Findings are context-bound to the participants Fewer men than women in the research.
Incerti et al. [26]	Australia	Grounded theory of qualitative interviews	In-depth interviews using semi-structured interview guide, with 13 sisters of substance abusers, using purposive sampling method.	To address significant gap, and draw upon DeFrain’s (1999) six qualities of a strong family to answer the research question: “Does a person’s problematic substance use impact upon their sibling relationships? “	+ +	Small sample size The findings cannot be generalized to the wider community.
Barnard et al. [27]	Scotland	Deviant case analysis of qualitative interviews	Semi-structured interviews with: 24 drug users, 20 parents, 20 siblings, 10 experts	To explore influence that a problem drug-using sibling might exert on the initiation of a brother or sister into drug use and how parents might respond to such a threat and the ways in which families adapted in response to family members who developed drug problems.	+	Small sample size
McCann et al. [28]	Australia	Interpretive Phenomenological Analysis of a qualitative interviews.	31 Semi-structured interviews with Addiction Affected Family members using the Purposive sampling method (14 parents, 13 couples, 4 siblings)	To explore the experience of AFMs who support a close relative with AOD misuse	+ +	The findings are context-bound Participants were predominantly females.
Hoeck and Van Hal [29]	Finland	Thematic analysis of qualitative interviews	12 in-depth interviews with parents of substance abusing young people	To explore experiences of parents of substance-abusing young people attending support groups regarding several topics related to the substance-abuse of their son or daughter, the impact on their lives and their views on social support.	+ +	The small group of parents interviewed The majority of the participants were women. The type of drug abuse was only reported by the involved parent.
Rodrigues et al. [30]	Brazil	exploratory, analytical, qualitative and comprehensive study	The participant observation of the family groups and 15 In-depth interviews with family members	To understand family members’ feelings about drug addiction.	+	Having at most two family members as respondents, due to the difficulty of contacting the whole group. Not adding the experiences of the drug dependent himself.
McCann and Lubman [31]	Australia	Interpretive Phenomenological Analysis of a qualitative interviews.	Semi-structured interviews with 31 Addiction Affected Family members	to understand affected family members (AFMs)’ experience of stigma within the context of substance misuse, and to explicate what steps, if any, they took to try to counteract stigma and social isolation.	+ +	Different stigma experience between AFMs involved in peer support groups, or those whose family member is in recovery.
Wilson et al. [32]	Australia	Thematic analysis of synchronous online counselling transcripts of partners	100 synchronous online counselling transcripts of partners of individuals with problem alcohol or other drug use	To explore the breadth of interpersonal impacts on a broader range of partners, to better inform service provision.	+ +	The number of male transcripts was lower than women. The data were sourced from online counselling transcripts, therefore, the findings may not be representative of the interpersonal impacts partners would seek help for in a face-to-face context.
Ólafsdóttir [33]	Iceland	Mixed-methods and Phenomenological Analysis of qualitative interviews	16 semi-structured interviews with four spouses/ partners, four parents, four siblings, and four (adult) children of individuals with substance abuse problem	To explore how family members of individuals with substance use disorder (SUD) experience its effect on the mental health and psychosocial state of other family members and the family system	+ +	Small sample size All participants shared a willingness to participate in family group therapy. It was the only researcher to carry out all of the interviews, analysis, and interpretation of the data.
Arlappa et al. [8]	India	Case Study with An Exploratory Analysis	10 households case studies with drug-dependent members and Focused Group Discussions with the youth and women of the concerned families living and 10 Semi-structured interviews with them	To explore the impact of addiction in a family	+	No discussion of limitations
Salter and Clark [34]	England	Grounded theory of qualitative interviews	10 Semi-structured interviews with parents of drug users.	To conduct a detailed qualitative analysis into the impact of substance misuse on the family, from the point of view of parents of drug users	+ +	Small sample size A huge variability exists between families. The treatment that participants in the sample had received is likely to have influenced their experiences in some way.
Groenewald [35]	South Africa	Case Study with Interpretive Phenomenological Analysis	A case report of a mother coping with an adolescent who has a drug use problem	To explore lived experience of a mother with an adolescent drug abuser	+	No discussion of limitations
W. Choate [36]	Canada	Grounded theory of qualitative interviews	Semi-structured interviews with adult caregivers of 21 teenagers with alcohol or drug abuse problem	To explore the parental perspective as they attempted to adapt and cope with substance dependency in their teenage children	+ +	The siblings have not been interviewed directly. Less intense interventions have been successful with a similar population Using a convenience sample.
Jackson et al. [37]	Australia	Thematic analysis of qualitative interviews	In-depth interviews with 18 parents of drug-abusing young people	To develop understandings into the effects of adolescent drug use on family life	+ +	Small sample size. The sample was limited to people who could read and converse fluently in English.
Usher et al. [38]	Australia	Phenomenological hermeneutic approach with Thematic analysis of qualitative interviews	In-depth interviews with 18 parents of adolescents with substance abuse problem	To describe and construct an interpretation of the lived experiences of parenting an adolescent who abuses illicit substances	+ +	The recruitment method (parents who did not respond to the media campaign may have a different story to tell).
Orford et al. [39]	England- Mexico	Grounded theory of qualitative interviews	24 Semi-structured interviews with 12 English and 12 matched Mexican family members	To explore experiences of stress and pressure in affected family members in England and Mexico and discovering the cultural differences between them.	+	Small sample size. Few male transcripts Few partners transcripts.
Bulter and Bauld [40]	England	Framework analysis approach of qualitative interviews	22 Semi-structured interviews with parents of heroin users and staff from a support agency that worked with families affected by drug use	To explore the role of the organization in supporting families affected by drug misuse	+	The sample was not random. The recruitment method.
W. Choate [41]	Canada	Qualitative review of qualitative interviews (content analysis)	Semi-structured interviews with 31 parents or caregivers of 21 adolescents	To explore the process that parents experienced, how they sought intervention and the ways in which support systems aided or hindered	+	Using a convenience sample. The siblings have not been interviewed.
Groenewald and Bhana [42]	South Africa	Interpretive Phenomenological Analysis of a qualitative interviews.	In-depth interview and 5 case reports of a mothers of adolescents troubled by substance abuse	To explore mothers’ experiences of living with an adolescent with substance use problems	+ +	No discussion of limitations
Velleman et al. [43]	England	Qualitative and quantitative analysis of qualitative interviews	Semi-structured interviews with 50 close relatives of identified problem drug users	To describe the various experiences to which family members told us they had been exposed; and to describe the various effects to which these experiences led.	+	Limited discussion of limitations
Masombuka [44]	South Africa	Explorative, descriptive and contextual research design	8 Semi-structured interviews with parents of children with addiction problem	To explore parents‟ experience and support needs with regard to their children’s addiction to nyaope	+ +	Small sample size. The recruitment method (Only parents who reached out for help, only parents who were conversant in English, Setswana) Limiting the gender perspective (Few male transcripts).
Jackson and Mannix [45]	Australia	Exploratory-descriptive study	Conversational style interviews with 12 mothers	To explore the perspective of mothers of adolescent cannabis use	+	The recruitment method (only Anglo-Australian middle class women living in two Australian states)
Arcidiacono et al. [46]	Italia	Grounded theory of qualitative interviews	Semi-structured interviews with 113 family members (FMs) of people with serious alcohol or drug problems	To examine the impact on Italian family members of living with a relative who had an alcohol or drug problem	+ +	No discussion of limitations
Ahuja et al. [47]	England	Grounded theory of qualitative interviews	Semi-structured interviews with 24 British Sikh wives of men with identified alcohol problems, plus 10 of their husbands and 7 of their daughters	To explore experiences of wives and daughters of people with alcohol abuse	+ +	The lack of control over sampling. Small sample of substance abuser and children.

### Data extraction

The data of the determined studies were extracted using a standard format for the systematic review of qualitative research (the National Institute of Health and Care Excellence [NICE] 2102a) [48]. The characteristics identified from each study included research questions, methods, sample size, and quality assessment. The desired data were carefully extracted and organized in relevant tables. In order to synthesize and analyze the findings of the studies, the desired data from the studies were extracted and categorized into specific thematic categories. Subsequently, the patterns present in the findings of the studies were searched and scrutinized to achieve a better and more comprehensive understanding of the issues and problems caused by addiction toward families.

### Quality assessment

The quality assessment of the articles extracted from the journals was performed using the NICE quality assessment checklist for qualitative research (NICE 2012b) [49]. By following this guideline, the conducted assessment investigates the research questions and the robustness of the method concerning the key findings and a valid conclusion. Six main areas are considered and assessed in this guideline: Theoretical approach, study design, data collection, data reliability and validity, analysis method, and ethical considerations. The seventh overall assessment deals with the relevance of the study and provides an overall rating: “ +  + ” in cases where all or most of the checklist criteria have been met and in cases where they have not been met but the conclusion has not been affected; “ + ” where some of the checklist criteria have been met and the conclusion is unlikely to change; and “-” where few or no criteria are met.

Two studies have been included in this systematic study using a combined method, and the quality assessment was performed only on the results of the qualitative data methodology of these studies. Regarding the quality assessment of the present study, a reviewer-researcher initially assessed the quality of the included studies, and the quality assessment was subsequently confirmed by another reviewer. Each search process in the different databases, the initial review of the found documents, matching with the inclusion and exclusion criteria of the findings, and quality assessment were conducted by two researchers independently to increase validity. In this study, the researchers were committed to being sufficiently accurate and honest in using the different sources in all stages of the work, including data collection, data analysis, and the report of the findings. The intellectual rights of all individuals related to the research are fully respected.

## Results

This study identified 99 abstracts screened for relevance to qualitative studies on addiction-affected families. Fifty-three full-text articles were studied and assessed, and 25 studies were finally identified as suitable for this review study. The obtained results were organized in relevant tables and classified into specific groups.

### Characteristics of target studies

#### Sample

Of the 25 studies in the systematic review, only one analyzed the transcripts of online interviews, and the other 24 studies were results of direct contact with the target groups. The total number of participants in the studies included in this systematic review was 728 people, among whom parents (*n* = 288, 39.56%), couples (*n* = 222, 30.495%), sisters/brothers (*n* = 65, 8. 92%), relatives (*n* = 63, 8.65%), drug users (*n* = 39, 5.35%), children (*n* = 21, 2.88%), specialists (*n* = 20, 2.74%), and female heads of households (*n* = 10, 1.37%) accounted for the largest percentage of participants in the target studies of this research respectively. Also, the number of samples in the studies shows a great variety, from the smallest number of samples in a case study (*n* = 1) [35] to the largest number of samples in a study (*n* = 113) [46].

#### Geographical characteristics

In terms of the geographical distribution and information of the studies included in this systematic review, there are 8 studies conducted in Australia (32%), 4 studies from England (16%), 3 studies from South Africa (12%), 2 studies from Canada (8%), 1 study as a result of collaboration between England and Mexico (4%), and 1 study each for Scotland, Indonesia, Finland, Brazil, Iceland, India, and Italy (4% each).

#### Quality assessment

The quality assessment carried out according to the NICE guidelines among the studies identified for the present research showed that 16 studies were of high quality (64%) and 9 were of medium quality (36%).

#### Qualitative methods

The most common qualitative methods used in the target studies reviewed in the present study included 7 studies using ethnographic and phenomenological methods (28%), 6 studies using the grounded theory method (24%), 3 studies using the thematic analysis method (12%), the use of the descriptive-exploratory method in 2 studies (8%), and 2 studies using the content analysis method (8%). In the remaining 5 studies (20%), various qualitative methods have been used, including 1 case using deviant case analysis in a study in Scotland with 74 samples of relatives, parents, and experts of the drug abuser [27], 1 case using the qualitative-analytical-exploratory method [30], 1 case study of 10 female heads of families [8], 1 case study of a mother with a child with drug abuse that has been analyzed using the interpretive phenomenological method [35], and 1 case using the framework analysis approach [40].

#### Thematic analysis of results

**Fig. 2:**
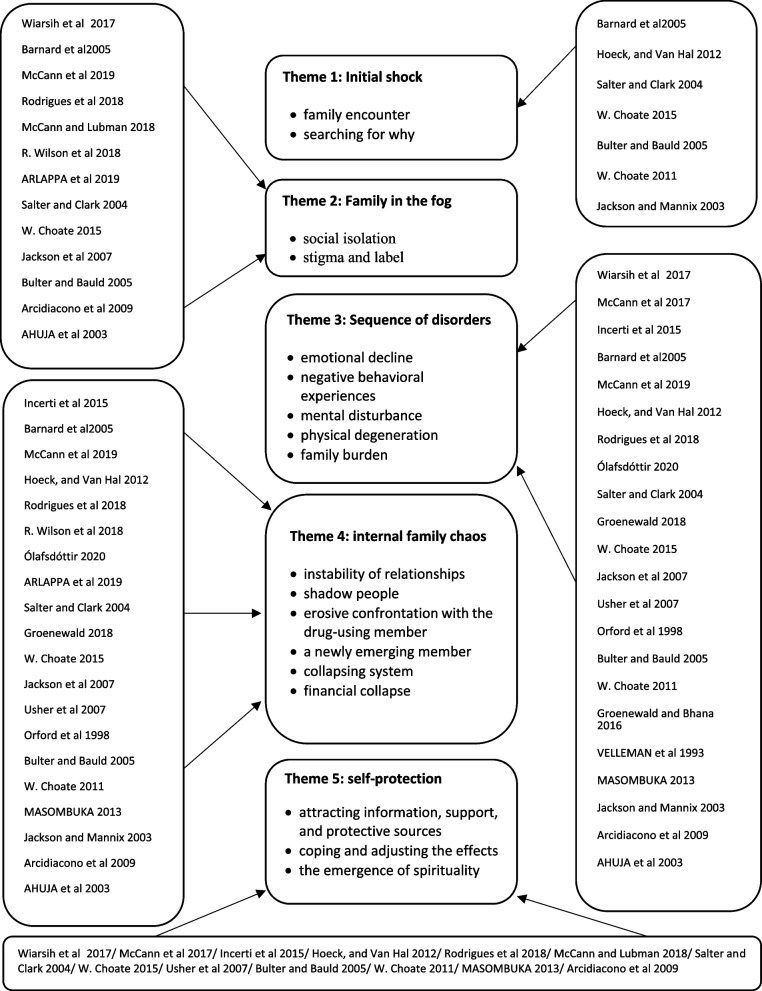
Diagram of identified themes

In order to analyze primary qualitative data, the thematic analysis approach is often used [50]. This approach is also an applicable method which can be used to synthesis the findings of multiple qualitative studies [51]. The synthesis will surpass the content of the original studies and generate further conceptions or understandings through developing the analytical themes [51, 52]. The following three stages of thematic synthesis, planned by Thomas and Harden [51], were used: (1) Free line-by-line coding of the findings of the primary studies, (2) the organization of ‘free codes’ into related areas to construct descriptive themes and (3), finally, the development of analytical themes. In this synthesis, the published results from each of the included studies were coded. A thematic analysis of the findings of the studies identified 5 main themes: 1) Initial shock (family encounter, searching for why), 2) family in the fog (social isolation, stigma and label), 3) sequence of disorders (emotional decline, negative behavioral experiences, mental disturbance, physical degeneration, family burden), 4) internal family chaos (instability of relationships, shadow people, erosive confrontation with the drug-using member, a newly emerging member, collapsing system, financial collapse), and 5) self-protection (attracting information, support, and protective sources, coping and adjusting the effects, the emergence of spirituality). The visual outline of the identified themes can be seen in Fig. 2. The frequent themes identified in the articles include the sequence of disorders in 22 studies, internal chaos in 18 studies, self-protection in 13 studies, family in the fog in 13 studies, and initial shock in 7 studies (Fig. 2).

The first theme (initial shock) is one of the themes identified from analyzing the findings of the studies consisting of themes such as family encounter and searching for why. Confusion, anger, inability, and dysfunction due to the family’s initial effort to respond to the raised problem are among the things that are mentioned in Barnard’s study [27], which uses the deviant case analysis method. Also, in a study to investigate the experiences of parents, Hoeck [29] investigated how parents are informed of their children’s addiction (being informed by a third party (police or hospital)/ by the drug user and the parents themselves) and showed that the families’ knowledge about addiction was very little at first or they had no information about it and there was no free discussion to express such problems in the home environment. Another study by Salter et al. [34], using the grounded theory method in England, showed that families suffered from difficulties such as confusion in the initial encounter with a problem and experienced problems like lack of awareness, the gradual process of awareness, drug user’s deceptive behavior, denial, self-contradictions, and the effects of external factors in the initial encounter with the addiction problem of one of their members. Investigating the process of parents’ adaptation and coping with a drug-abusing teenager was one of the goals that Choate [36] sought in a study using the grounded theory method. The results of the mentioned study indicate that the family first faces the way of becoming aware of their child’s drug addiction and perceives it as an intensified and progressive problem and then tries to find a logical answer for the cause and reasons for such a problem. At the same time, many parents proceed with examining their own behavioral records, and some parents consider their drug use experiences as a trigger and factor for their child’s drug addiction. In a study conducted using Ritchie and Spencer’s framework approach, Bulter et al. [40] viewed the family’s way of dealing with the addiction of one of its members as being one of the following cases: A person’s voluntary confession of drug use, observing abnormal behavior or changes in their normal behavior, denial of their drug use, difficulty recognizing, family devastation because their child is using drugs, experiencing it as a very shocking and traumatic event, experiencing it as the greatest fear, feeling defeated, self-blame, and the feeling of great shame at the beginning of facing the addiction problem. It is worth mentioning that the determination of factors and reasons such as mental health problems or learning problems as triggers and factors of drug dependence were among the explanations and mechanisms mentioned by the participants of Choate’s study [41] about the initial encounter with the addiction problem. The analysis of Jackson and Mannix’s [45] findings showed that one of the themes about which the addiction-affected families expressed their experiences was initial exposure and awareness. In this descriptive-exploratory study, the participants described their initial awareness of their child’s addiction with the sentence, “I could not deny that this had happened.” Generally speaking, the initial exposure and the way of awareness and then trying to respond to the addiction problem are among the negative experiences to which addiction-affected families frequently pay attention.

The second theme (family in the fog), with themes such as social isolation and stigma and label, is among other themes identified by qualitative analysis of the findings of the target studies. Regarding the stigma and label theme, in a study conducted via the phenomenological method to investigate the experiences of families with drug-using children, Wiarsih et al. [24] considered the family’s labeling one of the results of having a drug-abusing child. The findings of the mentioned study show that society’s attitude and feedback, self-view, and social discrimination are among the things that families perceive under the title of stigma. In this regard, in a qualitative-analytical and exploratory study on addiction-affected families, Rodrigues [30] has viewed society’s prejudice and society’s experience of stigma by the families to be of the factors that change the lives of families with drug-using members. The experience of stigma as one of the stressful factors at the level of families’ extended interactions is one of the findings of a study by Salter [34] in England using the grounded theory method. Among other themes identified in the present study is the social isolation of addiction-affected families. One of the negative experiences of addiction-affected family members is inhibiting social activities and, in some way, being away from the society and being isolated. Limited activities and social roles, especially for those with the most supportive contact with the drug user, were one of the findings of McCann’s study [28] conducted in Australia. In addition, in another study carried out to investigate the experiences of families regarding dealing with social isolation, McCann [25] showed that one of the families’ measures in dealing with the addiction of their family members was to minimize contact with others and consequently face things such as shame and embarrassment, fear of being judged by others, self-quarantine, and unwillingness to access informal and official support. In this regard, in a study carried out by the thematic analysis of 100 transcripts of online interviews with couples, Wilson [32] presented the impact on the family social network and the challenges of families in this area as one of the main themes of his research. In another work of research by Arlappa et al. [8], which was conducted in India as a case study, the effects of a drug-using member on a family’s social life were presented as one of the results of the study, according to which families prefer to limit their social interactions following the addiction of one of their members. In a study to examine the effects of addiction on family from the parents’ perspectives, Salter [34] analyzed the data obtained from semi-structured interviews using the grounded theory method. The findings of the mentioned study show that addiction-affected families are disturbed in the broad and essential levels of their interactions, which will result in the disassociation of such families from the social world and lack of active participation by those families. Also, many families will face the challenge of getting help because they try to hide the addiction problem of one of their members and thus limit their communication and experience serious challenges regarding asking for help and support [36]. It should be mentioned that the challenge of asking for help and the weak support of addiction-affected families are due to hiding the problem from others and disassociating from others, which is also one of the findings of Arcidiacono’s study [46] in Australia. Isolation of the family is another finding in this section, to which Jackson [37] refers in the findings of his study in Australia as the main theme of isolated, disgraced, and humiliated as if the family is only with the drug user and has no other social connection. This concept is also expressed in the results from Ahuja’s study [47] under the title of social isolation and also in the results of Bulter’s study [40]. The results from Bulter’s study indicated that self-imposed experiences, the family’s feelings of embarrassment or shame due to drug use by one of their members, and worrying about others’ opinions played a significant role in the isolation of family members and parents.

The third theme (sequence of disorders) is completed with themes such as emotional decline, negative behavioral experiences, mental disturbance, physical degeneration, and family burden. The findings of the present study showed that family’s emotional dimension is severely affected due to the abuse of one of its members, and families experience a wide range of emotionally distressing situations. The feelings of disbelief, non-acceptance, disappointment, shame, shock, anger, and regret in the family are mentioned in the findings by Wiarsih et al. [24]. Furthermore, the findings of Incerti’s study [26] have indicated feelings of sadness, hopelessness, and distrust as the fundamental challenges against addiction-affected families. Fatigue and emotional exhaustion, as well as fear and despair about the future in addiction-affected families, were mentioned in the results of an interpretive phenomenological analysis of 31 semi-structured interviews in McCann’s study [28] in Australia. Feelings of helplessness and despair, desperate cries for help, and living with guilt are feeling which are typical examples of the emotional distress experiences of addiction-affected families, which were found by various studies [29, 35, 38, 41, 44]. Families naturally experience a wide range of emotions, a significant part of which are positive emotions. However, in his study to investigate the experiences of addiction-affected families in Iceland with 16 participants, Ólafsdóttir [33] showed that one of the negative experiences of addiction-affected families was the transformation of their positive feelings, such as worry and care, over time to negative emotions, such as anger, shame, and sadness. Also, the increase in negative feelings toward the drug user, the increase in negative feelings caused by the drug user, and the increase in negative feelings in general (confusion, etc.) in Salter’s study [34], emotional distress and the use of pills to moderate stress in Groenewald’s study [35], and variable emotions shifting from positive components (love, admiration, and care) to negative components (desire for separation, etc.) in Velleman’s study [43] are among other findings in this regard. By analyzing the findings of the target studies, it was found that negative emotional experiences in families with addiction problems were widespread. In addition to the mentioned cases, there were cases such as worry and uncertainty about the individual’s situation, the family’s status, the family’s future, the effects of the individual on the family [34, 43, 46], loss of trust and feeling of mistrust [37, 46], feelings of blame and shame [37, 38, 41, 44, 46], feelings of fear and trembling of family members [44, 45], feeling of sadness due to the drug user’s negative and unexpected path [38], the emergence of destructive emotions such as inferiority, anger, division, and separation [39], negative effects on individuals’ emotional health, and the emergence of a wide range of negative emotions from continuous crying to the feeling of separation and leaving the family [40]. Negative behavioral experience is one of the other themes identified in the present study. Experiencing aggression and verbal or physical violence [25, 27, 29, 33, 37, 46, 53], theft [27, 30, 41], tension and controversy at home [34], and experiencing domestic violence towards household members and items [47] are among the cases mentioned by addiction-affected families in the target studies. Additionally, in the psychological dimension, the families reported negative experiences under the title of mental disturbance. Things like different levels of stress and anxiety [27, 29, 33, 53], the experience of mental and psychological violence [33], different effects on mental health [34], and suicidal ideation and attempt [35] are among the cases identified from the analysis of the findings of the target studies. The findings from Orford’s study [39], which was carried out in order to investigate the experiences of stress and pressure in addiction-affected families, indicated that the physical health of families was also affected by the addiction of one of their members, and family members sometimes reported cases such as the emergence of physical weakness, specific physical symptoms, excessive fatigue, lack of sleep, and anorexia. The incidence of physical degeneration was also identified and investigated in the findings of other studies [34, 40]. In addition to the mentioned cases, families also have an unpleasant experience under the title of family pressure and burden as a result of the drug abuse process of one of their members. The analysis of the findings of the studies showed that physical, psychological, social, and economic burdens [24], high levels of tension and pressure during the treatment of the drug-using member [34], and long-term processes of treatment and rehabilitation [36] were among the things experienced by addiction-affected families in this regard.

The fourth theme (internal family chaos) was another identified theme completed with the following themes: Instability of relationships, shadow people, erosive confrontation with the drug-using member, a newly emerging member, collapsing system, and financial collapse. The internal relations of families are among the first things which are affected by the newly created conditions, and families experience a wide range of disorders in this area, from problems and differences between parents and extensive marital differences [8, 29, 47] to the disturbance of the general structure of interpersonal relationships at home [27, 30, 32, 34, 40]. Also, the occurrence of chaos and failure in family communication and family conflicts are other things mentioned in the findings of the studies [41]. “Shadow people” refers to family members who have been neglected by others, especially parents, due to the addiction of another member, and the needs and psychological conditions of these individuals are somehow not paid attention to. Being neglected [34] and being ignored and not approved [26], being exposed to drugs and the possibility of entering the use process and dealing with public reactions [27], experiencing negative psychosocial effects, being isolated [33], suppressed anger and rage [37], lack of affection and attention, as well as reluctance towards social interactions and bringing friends to the home environment [47] are among the issues mentioned in various studies. In dealing with the drug user, the families also tried different ways, and the analysis of the findings showed that a significant part of the families’ energy is spent on these efforts. Rejection of the drug user was one of these ways that Barnard [27] found in his study in Scotland, and Orford [39] referred to it in his study as “an unpleasant life with the drug user.” Controlling the person and setting various limits for the drug-using person were other ways used by the families to deal with their drug-abusing member. Worrying about the person, trying to protect the person, and maintaining the relationship with love and friendship were other actions by the families in this area [38, 45]. Also, the participants of Arcidiacono’s study [46] stated that the person was good but had terrible abuse, and with this view, they confronted their drug-abusing member as if a new person had emerged who had nothing in common with the previous one. The new person was a person whom the family did not trust, had not been at home in general as if he/she was missing, had left the house without any notice and the time of their return was not known, did not take any family rituals and gatherings (birthdays, Nowruz, etc.) seriously and was absent in them, had a noticeable inability to respond to the family’s expectations [43], their friends had changed in general, and they suffered a sharp drop in education, as well as a decrease in personal hygiene and physical health [44]. It is worth mentioning that due to these issues, families tend to have serious problems in family economy and financial capability and experience a kind of financial collapse [8, 28, 30, 33, 34, 39–41, 46, 53]. Facing harmful family dynamics [28], threatening family functioning [29], jeopardizing the family system’s health[8, 33, 40, 44–46, 53], and moving from cohesion to confusion and collapse [46] are other threats that put the family on the path to internal chaos.

The fifth theme (self-protection) is related to the actions that families have taken to deal with new conditions. Themes such as attracting support sources, coping and adjusting the effects, and the emergence of spirituality are included in this category. In his study, Wiarsih [24] showed that the families tried to cope with the problem by attracting moral, financial, informational, and social support. In order to attract support sources, the findings of various other studies were also considered in this research. These findings showed that the attraction of support, informational, and therapeutic sources had been one of the dominant methods of encountering the addiction problem in the addiction-affected families studied [26, 29, 34, 40, 44, 46]. Among other actions of addiction-affected families to protect themselves are coping and adjusting the effects, such as problem-solving methods [24], dealing with violence [25], adjusting the effects of stigma [31], and various coping strategies to reduce the consequences of the addiction of a family member [29, 34, 38, 41, 44, 46]. Also, Rodrigues [30] showed in his study that faith and trust in God was one of the methods used by families to manage the effects of the problem and deal with it.

## Discussion

Although qualitative research on the issues and problems of addiction-affected families are limited and carried out in minority, these studies have been necessary to improve the understanding and insight of policymakers in this field, social service providers, professionals, and addiction-affected families. Effective and specialized support for this group is possible only when their voices are heard, and services are tailored to their conditions, and needs are noticed and provided for. For this purpose, this systematic review was conducted to identify and review those studies which have investigated the effects of addiction on addiction-affected families using qualitative research methods. The findings of the studies showed consistent themes among the research methods and the studied populations. Twenty-five high-quality and medium-quality articles with diverse contents which were suitable for the purposes of the current research were identified and reviewed. The analysis of the findings of the studies showed 5 main themes related to the fundamental challenges of addiction-affected families. The identified themes were the initial shock, family in the fog, sequence of disorders, internal family chaos, and self-protection. The initial encounter with addiction was one of the first themes of the present study. Families are initially confused and shocked due to lack of knowledge and experience [27, 29, 34, 40] and are somehow unsure of their next steps to take. Some families start self-prescription and take actions that they consider appropriate, which creates the background for future problems for family members. At this stage, the family puts itself in a deep, long, and recurring mourning process [24]. This concept is very specific and has been mentioned in very few studies. In other words, encountering a family member’s addiction for the first time is so painful that families refer to it as their hardest experience in the addiction process [54]. Generally, Placing families on educational grounds and introducing support groups can play a significant role in getting families out of this vicious cycle. These groups play a significant role in modulating the effects of the initial shock in addiction-affected families by providing information about addiction and treatment, strengthening the morale of addiction-affected families, providing support, understanding their needs, providing a learning contexts, teaching family members to distance themselves from problematic situations, helping them overcome feelings of guilt, shame, and failure, teaching them to deal with risks and fears, and teaching coping strategies (physical and emotional distance of the family from the drug-abusing person). Furthermore, the key sentence of support groups for families is: Learn to live with anxiety and fear [29].

The analysis of the findings showed that families opt for social isolation to avoid social stigma and being labeled. These two processes have been described in the present study with the second theme, i.e., family in the fog. The most important challenge that these families experience at this stage is the challenge of getting help [36], because they generally pose an unwillingness to access formal and informal support available in the society [31]. This action of the families is taken due to the defense mechanism of secrecy. Families somehow prefer to respond to the problem on their own in any way possible in order to avoid the possibility of judgment [31], stigma [34], and being labeled, so that they can avoid social discrimination against the family [24]. In this regard, some families move toward social isolation and some try to manage the effects of this problem by adopting measures such as adjusting the effects of stigma, challenging the misconceptions of the people around them about drug abuse, or choosing specific individuals and communicating with them [31]. Furthermore, the experiences of shame, stigma, and social isolation are among the results which Di Sarno et al. [17] found in their study, which was conducted via the scoping review aiming to investigate the mental and physical problems faced by addiction-affected families. In their study, in addition to quantitative studies, they also aimed at those qualitative studies which specifically focused on the mental and physical challenges against addiction-affected families and found the three above-mentioned challenges to be common among all the investigated qualitative studies. What is expected to be noticed by policymakers and service providers is to eliminate the misconceptions about addiction-affected families. Support groups should compile and implement effective measures to adjust and finally remove the effects of social stigma. Also, the development of effective interventions at the individual and social levels by researchers in this field aiming at removing the social barriers against addiction-affected families can play a significant role in preventing the social isolation of this at-risk group.

The third and fourth themes identified in this study indicate the extent of harm in the family, both at the family health level and the family system and functioning level. Concerning the various aspects of the health of addiction-affected families, studies focusing on this area provide insights into the consequences of damages to emotional, mental, physical, and behavioral health of families with a drug-abusing member, showing the sequence of damages for addiction-affected families. The concept above is among the concepts which have been mentioned in various studies. In other words, addiction deeply affects family members on the psychological, emotional, physical, and behavioral levels [55–57].

Furthermore, concerning the fourth theme, i.e., internal chaos, this study determined that the family system and functioning might face serious threats at the levels of relationships, as well as in the family health system. Conflicts within the families affected by addition are among the challenges which all family members have mentioned and considered to be an indispensable part of addiction. It is to be noted that improper construct and function of a family and morbid relations between family members cause them to face more severe challenges and pressures [13]. Since different levels of family health and function are influenced in the third and fourth themes and all of the reviewed studies have taken them into account, it seems necessary to take measures in order to alleviate their effects.

Specialists consider family a source and support for its members, who fulfill their duties with all their limitations [27]. The results of the investigations showed various interventions around the world with different goals for addiction-affected families, and their implementation can play a key role in helping families exit this Bermuda process. Increasing social support, coping skills, modulating stress and pressure [58], reducing the symptoms of mental disorders and improving family functioning [59], improving family functioning at the system, structure, and strategy levels [60], training families in the areas of emotional support, social acceptance, reduction of problems caused by addiction [61], awareness of family needs, environmental and interpersonal changes, organization of family structure, use of social strengthening and family education models (craft) [62] and Matrix [63], participating in Naranan meetings for addiction-affected families, and participation in meetings are only part of the existing interventions for promoting and improving addiction-affected families. It is worth mentioning that one of the things that the experts in this area emphasize is the necessity to pay attention to those individuals who are at home under the shadow of the drug-using person. In other words, families, especially parents, neglect others due to the problems caused by the addiction of one of their members and spend all the energy and internal resources of the family (financial, mental and psychological, social, and cultural) on the mentioned person. This issue creates severe problems in the long run for other family members due to daily encounters with these issues and facing addiction to such an extent that may cause these individuals to suffer from severe psychosocial problems and isolation [53] or enter the path of drug use [27]. Since this systematic review has targeted all the qualitative studies conducted in the field of addiction-affected families and has identified valuable and comprehensive themes, the need to develop a comprehensive intervention according to the data of the present study, taking all dimensions of families into account, has become ever more evident.

As the final theme identified, self-protection is the main key to starting the recovery process in addiction-affected families. Seeking help, moral support, financial support, informational support, and social support [24] and trying to deal with the problem and reduce its negative effects are parts of the process that addiction-affected families embark on for self-protection. It is to be noted that lack of social support exposes families to serious problems [13]. The final concept in this section is the recovery process of addiction-affected families, which starts when they face the addiction of one of their members and are somehow involved in its maze. Intervening in the levels of compatibility with the drug user’s behavior, financial compliance with conditions, hidden interventions, formal and informal support, religious and spiritual support [12], and preparing for changes in family members and family functioning and increasing coping skills [64] all play an effective role in providing a context for families to protect themselves and ultimately lead to the recovery of families.

In general, the point revealed in this study and mentioned in all reviewed studies was that when the families expressed the challenges caused by addiction, they also expressed their efforts to overcome the problems and called it the challenge that addiction had created for them. In other words, the families tried to survive and keep their family members alive while they were frustrated and exhausted and provided the basis for the family’s recovery. Moreover, it is to be taken into account that the experience of addiction varies for different families based on social and cultural conditions, and provided that there is proper social support and healthy family functioning, family members will face less serious challenges when having to deal with the addition of one of the members [13]. However, they had doubts about how to do it, and they were prone to confusion and ambiguity. Investigating the recovery process of families is not one of the goals of this study, but it is a topic that can be considered for future research and used as a guide, reference, and path for the recovery of addiction-affected families.

The method for the present study is the systematic review of qualitative studies in the area of experiences and challenges faced by family members affected by addiction. In order to analyze the data, a thematic analysis was used. This study was designed in such a way to analyze only those qualitative studies which comprehensively address the challenges which addiction-affected families face and possess the required standard in accordance with NICE quality assessment checklist for qualitative research. Among those studies which can be compared with the present study, one can mention the valuable study conducted by Di Sarno et al. [17], in which the researchers implemented the scoping method in order to investigate the mental and physical challenges faced by addiction-affected families. In that study, with respect to the aim of the study, all qualitative and quantitative studies which only focus on the mental and physical challenges against families affected by addiction are analyzed. However, the present study has adopted a more comprehensive approach in order to present, in addition to the concepts above, all the other dimensions of addiction-affected families and to use thematic analysis in order to put forward family challenges comprehensively in 5 different conceptual categories. The present study has taken a step beyond Orford’s review study [18]. Orford has presented a summary of qualitative studies conducted in the area of addiction-affected families until 2010 and reported the results in the four categories of stress, strain, coping, and social support. The present study, however, has analyzed all the research conducted until 2022 on the challenges faced by addiction-affected families and has presented 18 sub-themes in addition to its main 5 themes.

### Limitations and future directions of research

The present study was conducted using a protocol-oriented process and all the reliable scientific databases in the world. While, the concept of addiction includes a wide range of addictive behaviors, addiction in this research meant using any drug and alcohol. There are many limitations to the studies used in this research.

In the present study, the level at which a family member in engaged in substance use (low-risk drug users or occasional users compared to those users classified as harmful or dependent users) has not been considered, while this level can affect the challenges and difficulties faced by addiction-affected families. Three of the studies included in this review were published by the same authors and used the same group. However, each study addressed slightly different aspects of AAF’ experiences with living with a member with addiction problems [25, 28, 31]. It is important to mention that this study has not examined the families’ cultural, religious, and belief differences in dealing with addiction, and the lack of data and studies among different cultures and beliefs in this field is challenging for researchers. As a result, future studies should be able to show a better understanding of the psychosocial effects of addiction on families with more emphasis and sensitivity toward culture, ethnicity, and religion. Moreover, lack of attention to the role of the drug-using member in the family in the current study, can be effective regarding the type of its effects on other family members and other cases that can generally limit the conclusions that can be obtained from this study. For this reason, to produce more reliable results, future systematic studies should limit their search terms and phrases according to the role of the drug user in the family so that they can provide more reliable recommendations and suggestions to support addiction-affected families. Evidence shows that some studies, for various reasons, have more chances to be published in valid journals in the shortest time, and it is somehow easier to access and find such articles, while these articles may be poor in terms of both methodology and work processes. Therefore, in the current study, conclusions solely based on published studies can be misleading. The terms used in the field of addiction-affected families are diverse, and special titles and unique literature might have been used for some papers, as a result of which, we may have missed a number of relevant but inaccessible studies and future systematic studies should include a broader range of relevant terms to provide a more general insight and perspective regarding this group’s challenges and health status. However, in the present study, it is believed that the use of reliable and diverse scientific databases, double screening of the studies, and a strong search strategy have allowed us to identify all eligible articles. While this study has focused on the effects of drug addiction on families, it has also revealed the existence of a big gap in the knowledge of families affected by other addictive behaviors (Internet, gambling, etc.). It is, therefore, essential to compare and draw existing debates and narratives and their evolution over time to understand addiction-affected families’ challenges better.

In addition to the limitations outlined in Table 1 regarding the examined studies, there are other important considerations that warrant attention. Firstly, since the reviewed studies were qualitative in nature, the common limitation of "limited generalizability" applies to all the studies under investigation. In other words, the findings of the reviewed studies cannot be easily generalized. Furthermore, as most studies relied on help-seeking or service-receiving samples, and the selection of participants was based either on snowball sampling or purposive sampling, their experiences may have been influenced by the type of services they received, making them inadequate representatives for all families affected by addiction.

### Implications for research and practice

This is the first systematic review of qualitative research on the challenges of addiction-affected families, which has targeted studies over the past 30 years. Qualitative research provides an opportunity to hear the voices of research participants in order to provide valid empirical and perceptual evidence, which can be used to inform and influence policies and provide mental health services using an evidence-based perspective. This systematic review provides a reference of evidence-based knowledge obtained from qualitative research by drawing the themes and findings of qualitative studies on the challenges of addiction-affected families. The first outcome of the present study in practice can be to pay attention to wider dimensions (social, cultural, economic, and individual) of families. In other words, a drug-using person consciously or unconsciously faces their family with fundamental challenges, and these challenges provide the basis for future problems. For example, while societies try to accept this group, social stigmatization and labeling is an issue that this group constantly faces, and it plays a special role in concealment and self-censorship by families, which subsequently causes more severe problems to arise for them. The second outcome of this study for practice and action is the need to train social, educational, and health service providers in order for them to try to accept addiction-affected family members and provide psychosocial, educational, and preventive services in case of encountering any members of this group. Also, this review study has provided the basis for studies and interventions in this field, and, by providing a visual diagram of the identified themes, it has provided the framework for interventions needed by addiction-affected families for researchers in this field. It is worth mentioning that with access to these rich qualitative data, which were the results of the experiences of addiction-affected families, it is possible to design and implement more effective support and educational mechanisms for families that have just entered this long process.

## Conclusions

The analysis of the data obtained from the present research identified 5 main themes in this process: Initial shock, family in the fog, sequence of disorders, internal family chaos, and self-protection. The findings of the present study clearly state that any types of intervention to be carried out within addiction-affected families need to consider all problems and challenges created by addiction. These 5 themes were identified in different studies with different qualitative methods and different target populations. The implications of the present study at the levels of policymaking, practice, and research have also been clearly stated. Addiction-affected families want a space that is far from any judgment and labeling so that they can control their mental, psychological, and social conditions and the society can prevent the initial shock of this group when encountering the addiction of one of its members by arriving on time and providing the right educational services. The voices of addiction-affected families revealed the need for educational, informational, and therapeutic support to improve their coping skills in order to face and moderate the effects of addiction. The results of the present study provide a rich source of evidence-based information to provide the best services and policymaking for addiction-affected families. It is also important to mention that in developing countries and in countries where the governments play a small role in providing welfare for their citizens, individuals and families are intertwined elements, and any problem for each member can significantly impact the whole system. Therefore, paying attention to addiction-affected families in these areas is only in its initial stages, and the need to pay attention to this group has become apparent more than ever.

## Data Availability

All data generated or analyzed during this study are included in this published article.

## Abbreviations

AAF: Addiction Affected Family
FM: Family member
SUD: Substance use disorder
PSU: Problematic substance use
